# Activity Coefficients for Liquid Organic Reactions: Towards a Better Understanding of True Kinetics with the Synthesis of Jasmin Aldehyde as Showcase

**DOI:** 10.3390/ijms20153819

**Published:** 2019-08-05

**Authors:** Philippe M. Heynderickx

**Affiliations:** 1 Center for Environmental and Energy Research (CEER)—Engineering of Materials via Catalysis and Characterization, Ghent University Global Campus, 119 Songdomunhwa-Ro, Yeonsu-Gu, Incheon 406-840, Korea; Philippe.Heynderickx@ghent.ac.kr; Tel.: +82-32-626-4206; 2 Department of Green Chemistry and Technology, Faculty of Bioscience Engineering, Ghent University, 653 Coupure Links, B-9000 Ghent, Belgium

**Keywords:** modelling, liquid reactions, activity coefficients, parameter estimation

## Abstract

The aldol condensation of benzaldehyde and heptanal is taken as an example of reversible liquid phase organic reactions to show that inclusion of activity coefficients reveal distinct differences in conversion and product distribution when different solvents methanol, ethanol, n-propanol, or n-butanol are used. The purpose of this work is to show a pronounced solvent effect for a given set of identical kinetic parameters, i.e., the same liquid phase kinetics can result in different conversion and yield values, depending on the choice of solvent. It was shown that subsequent parameter estimation without inclusion of the activity coefficients resulted in a pronounced deviation from the ‘true’ kinetics, up to a factor of 30. It is proposed that the usage of average activity coefficients gives already a significant improvement, resulting in acceptable parameter estimates.

## 1. Introduction

Carbon–carbon bond formation reactions are paramount cornerstones in organic synthesis. One of the key reactions is the aldol condensation, which features the reaction of two carbonyl compounds to form a new β-hydroxy carbonyl compound under basic conditions [1,2]. The requirements for this reaction are that one of the reacting species must contain a protonated α-carbon adjacent to the carbonyl center and the proton attached to this carbon is acidic enough to be abstracted by a base to form a so-called ‘enolate’ ion. Upon reaction with a second carbonyl compound, the β-hydroxy carbonyl compound is formed, which can undergo elimination of water to form an enal compound. 

In order to scale up laboratory work, experimental concentration data can be used for parameter estimation in the absence of diffusion or gradients and these parameters can be used as input for simulation codes, which can investigate the optimal conditions for industrial practice. Hence, these kinetic parameters are of paramount importance for the description of the process. In this respect, it was noticed by the author that not many papers deal with the inclusion of the so-called ‘activity’ or ‘activity coefficients’ of the compounds. These values reflect the ‘true’ chemical concentration of compounds and, as such, they should be used in these parameter estimation procedures. Examples of applications of activity coefficients in parameter estimation can be found in references [3,4,5,6,7,8,9]. 

**Scheme 1:**
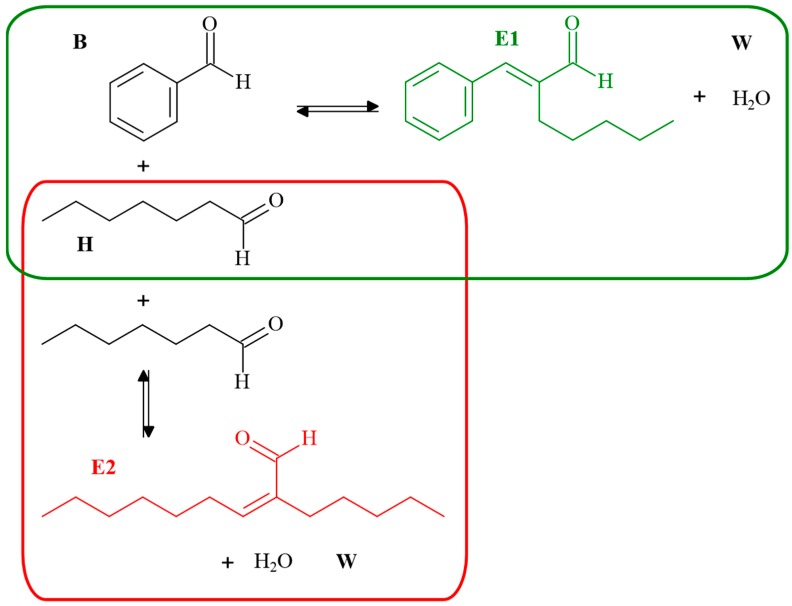
General scheme for aldol cross-condensation (green frame) and self-condensation reaction (red frame) with benzaldehyde (***B***); heptanal (***H***); jasminaldehyde (***E_1_***); water (***W***); and (**Z**)-2-pentylnon-2-enal (***E_2_***).

This work uses the reaction of benzaldehyde and 1-heptanal, which is an important aldol condensation reaction, in the production of α-pentyl cinnamaldehyde, as a test case. This fine chemical compound of jasmine odor is also known as ‘jasmine aldehyde’ and it is widely applied in the perfume industries [10,11,12,13]. Excellent experimental reports can be found in literature [12,14,15,16,17], see also Appendix A. The general reactions are shown in Scheme 1. 

Solvent effects on reaction yields and product distributions are well known in the field of organic chemistry [18,19,20,21]. With respect to aldol reactions, e.g., Kandel et al. reported a reversal of conversion profiles in the reaction between p-nitrobenzaldehyde and acetone with hexane or water as solvent [22]. Iglesias et al. reported differences in product distribution when different ionic liquids were applied in the aldol condensation of benzaldehyde and propanone [23]. Huang observed that the choice of solvent can also affect the specific product type of the aldol condensation, i.e., ethanol has beneficial effects on the aldol condensation, e.g., compared with tetrohydrofuran as solvent [24]. 

To this extent, four solvents (methanol, ethanol, n-propanol, and n-butanol) are used to simulate conversion and product distributions, starting from a set of kinetic parameters for the given aldol condensation reaction scheme. The main aim of this work is to give an answer to the question of whether chemical activity should be used rather than concentrations for appropriate parameter estimation. A practical ad hoc solution is presented, since the incorporation of activity coefficients might easily result in complex codes. Secondly, it is shown that, starting from the same kinetic coefficients for the given aldol condensation, different product distributions and conversions are obtained. The latter is indicating that sometimes different mechanisms are proposed for different solvents, but it might be that local activity coefficients change concentration dependencies, resulting in an increased or decreased contribution of certain reactions. 

## 2. Results and Discussion

**Figure 1:**
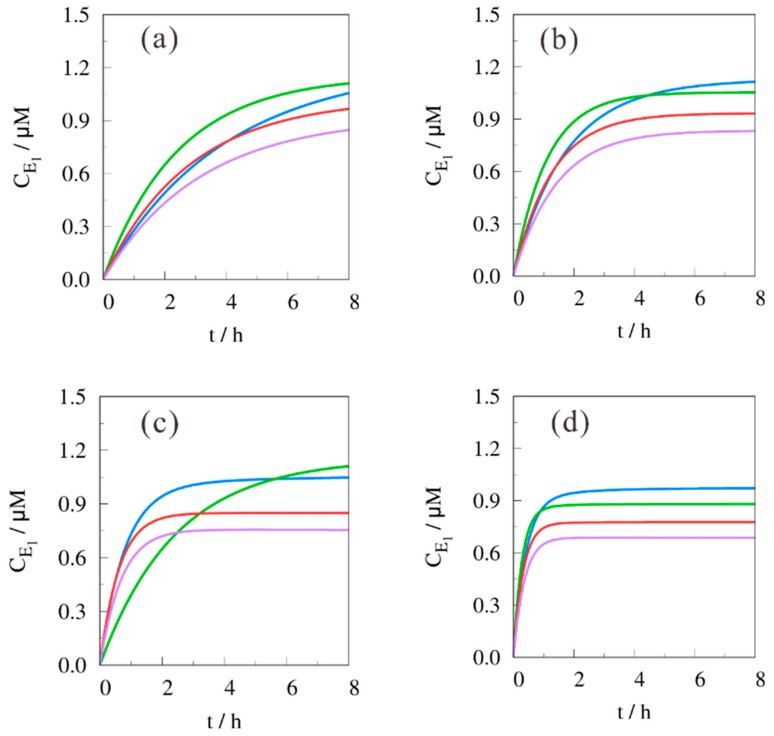
Concentration of jasmin aldehyde (*E*_1_) as a function of reaction time. (*B*/*H*)_0_ = 2 mol·mol^−1^, T = (**a**) 80 °C, (**b**) 100 °C, (**c**) 120 °C, (**d**) 140 °C; (**─**) methanol, (**─**) ethanol, (**─**) n-propanol, (**─**) n-butanol. Lines are obtained as described in Section 3.2.

Figure 1 shows the concentration of jasmin aldehyde as a function of reaction time for the four evaluated reaction temperatures and the condition (*B*/*H*)_0_ = 2 mol·mol^−1^. For the lowest temperature it can be concluded that ethanol was the best solvent, i.e., the highest jasmin aldehyde yield was obtained. For example, after 4 h of reaction the yield was 30% higher than in the case that other alcohol solvents are used. 

At 100 °C ethanol competes with methanol at higher reaction times, but overall ethanol is still a good choice in order to have the highest concentration of desired reaction product. For higher reaction temperatures, methanol and ethanol showed comparable results. Overall, it could be observed that n-propanol and n-butanol have the lowest yield for product *E*_1_, so it can be concluded that methanol or ethanol are advisable to be used in aldol condensation reactions. 

From Figure 1 it can also be deduced that equilibrium was faster reached for higher reaction temperatures. At 140 °C, equilibrium was reached after ~1 h, whereas for the lowest temperature the equilibrium was not established after 8 h. Regarding the calculation of the average activity coefficient, see Section 3.2, the subsequent spread will be more pronounced for lower temperatures, since the variation of the actual value versus reaction time was more pronounced. 

Based on the given simulations, a temperature of 140 °C is not advisable since *E*_1_ is obtained in concentrations lower than 1 μM. For the other (lower) temperatures, a concentration of ~1.10 μM is obtained. The author realizes that the difference is not that pronounced and that the results were in fact the consequence of the ballpark values for the kinetic parameters, but it is important to spot the relation between the temperature and equilibrium: The higher the former, the sooner the latter and, hence, no improvement in yield is possible. For the other initial conditions, (*B*/*H*)_0_ = 1 mol·mol^−1^ and (*B*/*H*)_0_ = 0.5 mol·mol^−1^, lower concentrations were obtained, so this is also a point for intensive process improvement. 

**Figure 2:**
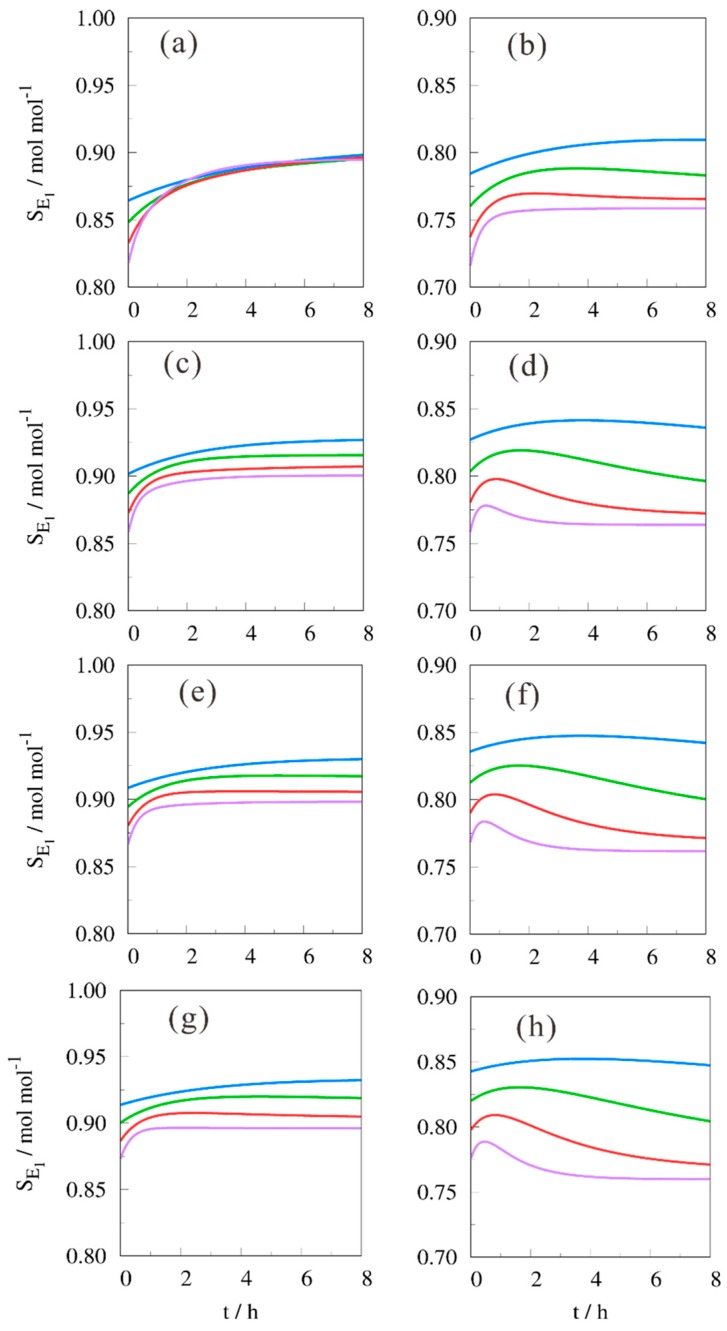
*E_1_* product distribution versus reaction time. Solvent = (**a**,**b**) methanol, (**c**,**d**) ethanol, (**e**,**f**) n-propanol, (**g**,**h**) n-butanol; (a,c,e,g) (B/H)_0_ = 2 mol·mol^−1^ and (b,d,f,h) (*B*/*H*)_0_ = 1 mol·mol^−1^; T = (**─**) 80 °C, (**─**) 100 °C, (**─**) 120 °C, (**─**) 140 °C.

Figure 2 shows the product distribution for *E_1_* versus reaction time. It was observed that a better selectivity was obtained for alcohols with a higher carbon number. However, the biggest difference was noticed for methanol versus ethanol. The application of n-propanol and n-butanol versus ethanol was a marginal gain and, therefore, possible spread on experimental data will probably result in very similar results. Again, it appears that ethanol is the best solvent for the given aldol condensation reaction, envisaging an optimal yield regarding jasmin aldehyde. 

It can also be recognized from Figure 2 that for an excess in benzaldehyde, (*B*/*H*)_0_ = 2 mol·mol^−1^, the product distributions were closer to each other, compared to an equimolar initial start of the experiment, i.e., (*B*/*H*)_0_ = 1. A second observation is that for the equimolar initial condition, a maximal value for the product distribution was obtained. For an excess of heptanal, (*B*/*H*)_0_ = 0.5 mol·mol^−1^, similar graphs were obtained, however at lower values for *S*_*E*1_, and, therefore, they are not shown in the manuscript. 

In this respect the reader is kindly reminded at this stage in the discussion of the results that, in case of not updating the activity coefficients, the same output would be produced. In other words, possible optimization points for maximal output were overlooked in that regard. This points out the importance of updating the activity coefficients with time, i.e., with varying composition of the reaction mixture. 

**Figure 3:**
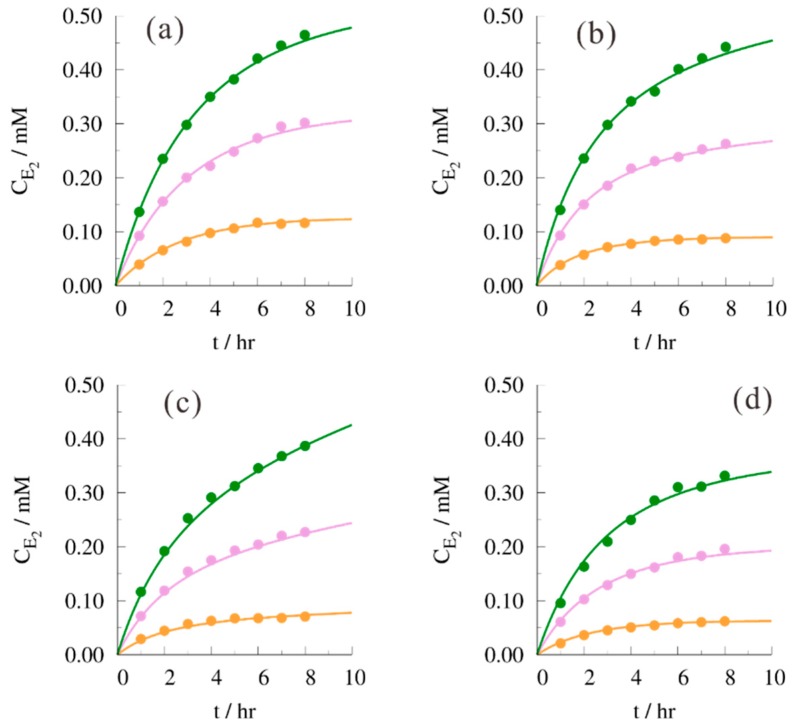
Concentration of byproduct E_2_ as a function of reaction time. T = 80 °C and solvent = (**a**) methanol; (**b**) ethanol; (**c**) n-propanol; (**d**) n-butanol; (**─**,**●**) (*B*/*H*)_0_ = 2 mol mol^−1^, (**─**,**●**) (*B*/*H*)_0_ = 1 mol mol^−1^, (**─**,**●**) (*B*/*H*)_0_ = 0.5 mol mol^−1^. Points and lines are obtained as described in Section 3.2.

Figure 3 plots byproduct E_2_ concentration versus reaction time at 80 °C. It can be clearly observed that for a higher carbon number of the alcohol as solvent, the contribution of the byproduct was less for the same initial conditions, and this perception was more pronounced for higher (*B*/*H*)_0_ values. For the sake of completeness, at higher temperatures similar trends were obtained as explained for 80 °C; however, higher E_2_ concentration values together with lower *E_1_* product distribution were noted. 

**Figure 4:**
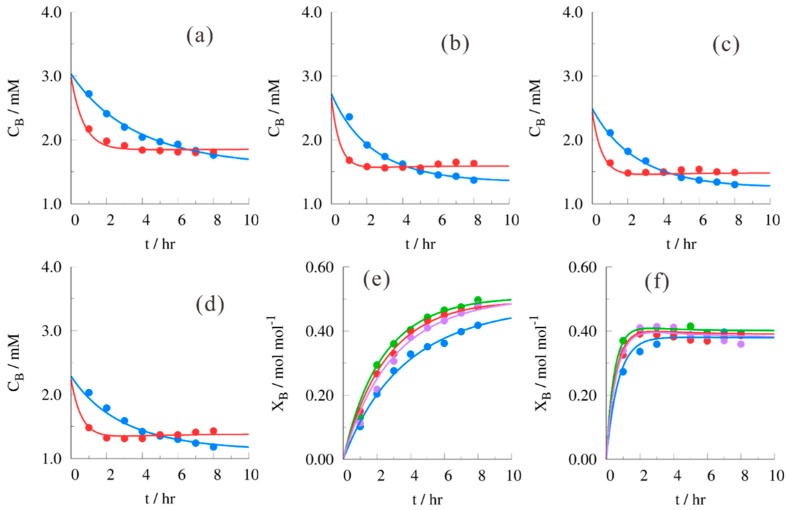
(I) Concentration of reactant B as a function of reaction time for (B/H)_0_ = 1 mol·mol^−1^. Solvent = (**a**) methanol; (**b**) ethanol; (**c**) n-propanol; (**d**) n-butanol; T = (**─**,●) 80 °C, (**─**,●) 120 °C. (II) Conversion of B as a function of reaction time for (*B*/*H*)_0_ = 1 mol mol^−1^ at (**e**) 80 °C, and (**f**) 120 °C with the solvent **(─**,●) methanol, (**─**,●) ethanol, (**─**,●) n-propanol, (**─**,●) n-butanol. Points and lines are obtained as described in Section 3.2.

Figure 4a–d describes the concentration of benzaldehyde versus reaction time for T = 80 °C and 120 °C. At higher temperatures a long reaction time did not result in an increase of *E_1_* production. Figure 4e,f depicts the benzaldehyde conversion and it is clear that methanol as solvent resulted in the lowest conversion results. At 80 °C, a conversion of ~0.48 mol·mol^−1^ was obtained for ethanol and the order was ethanol > n-propanol ≈ n-butanol > methanol, whereas at a higher temperature, e.g., at 120 °C, only ~ 0.37 mol·mol^−1^ was noted. In the latter case, the data overlap more or less, so a distinct ranking could not be made. 

**Figure 5:**
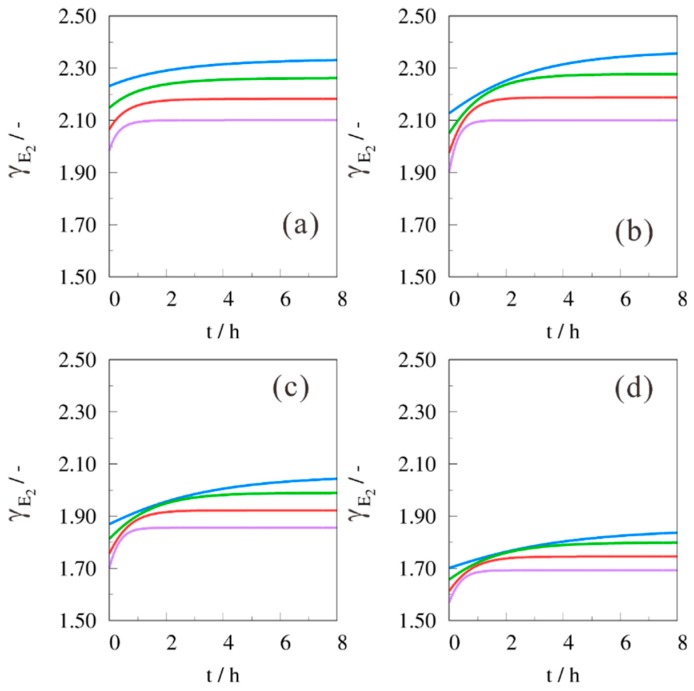
Activity coefficient for E_2_ versus reaction time with (*B*/*H*)_0_ = 2 mol·mol^−1^. Solvent = (**a**) methanol; (**b**) ethanol; (**c**) n-propanol; (**d**) n-butanol; T = (**─**) 80 °C, (**─**) 100 °C, (**─**) 120 °C, (**─**) 140 °C.

**Figure 6:**
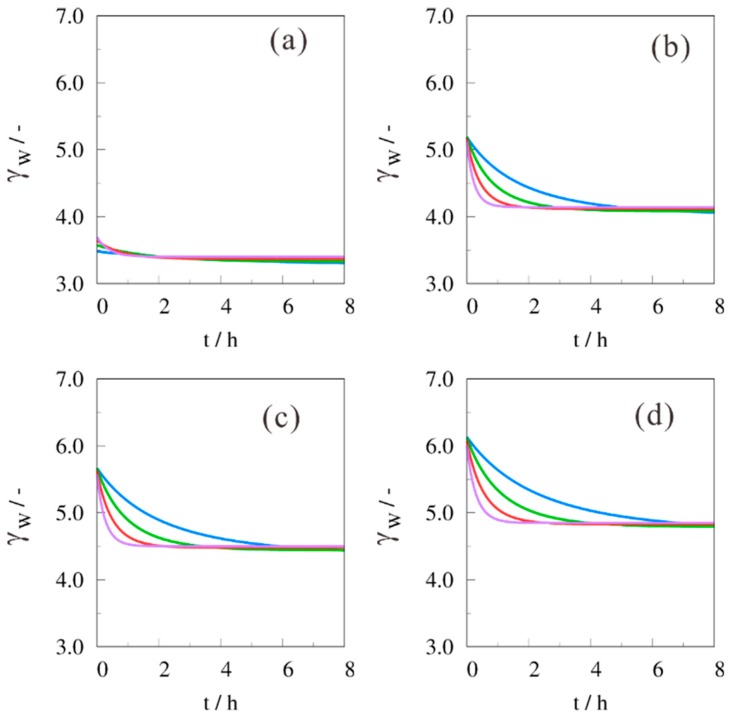
Activity coefficient for W versus reaction time with (*B*/*H*)_0_ = 2 mol·mol^−1^. Solvent = (**a**) methanol; (**b**) ethanol; (**c**) n-propanol; (**d**) n-butanol; T = (**─**) 80 °C, (**─**) 100 °C, (**─**) 120 °C, (**─**) 140 °C.

In the presented work, a lot of simulations were performed and in order not to overload the manuscript with data, only exemplary excerpts of activity coefficient results are reported. Figure 5 gives the activity coefficient for byproduct E_2_ versus reaction time with (*B*/*H*)_0_ = 2 mol·mol^−1^. The activity coefficient with methanol as solvent was ~2.20. This means that the ‘chemical concentration’ was 2.2 times higher than the physical concentration and this has a pronounced effect on concentration profiling or parameter estimation. It can also be spotted that for an increasing carbon number of the alcohol solvent, the activity codefficient decreased. This appears to contradict the previously made conclusions for Figure 4. However, inspection of Figure 6 reveals that the decrease in activity coefficient for *E*_2_, was compensated for by the increase of activity coefficient for W. In other words, the product of *a_E2_* and *a_W_*, both occurring in reaction rate (5), was increasing for an increasing carbon number in the alcohol solvent (not shown in the manuscript). This product influences the backward step in the equilibrium and, hence, this explains why for n-butanol a lower contribution of *E*_2_ was observed, compared to the use of methanol.

Regarding the temperature dependency of the activity coefficients, average values were plotted according to the following relation, lnγ = a + b/T, in order to check the value of b. In other words, if for example b was found to be 5 kJ·mol^−1^, the corresponding activation energy for the specific reaction would be estimated with at least a deviation of 5 kJ·mol^−1^, and the activity coefficients would appear together with the true kinetic parameters in the reaction rate expressions. The author is currently working on experimental data for which b values bigger than 5 kJ·mol^−1^ were found, having significant implications on the reported kinetics (details cannot be given, since the results are not yet published). In this work, however, the variations in activity coefficients versus temperature were not that pronounced, i.e., it was less than 1.5 kJ·mol^−1^, and this was just one of the reasons why this test case was developed: It can be expected that in the case of higher variations, the implications on the kinetic parameters and the corresponding parameter estimation procedure would be far more pronounced. This should be a caveat for future work. 

The product of activity coefficients, in the case of ethanol as solvent, are reported in Table 1, i.e., these values appear in the bimolecular reaction rates. It can be noticed that the correction for activity reached almost a factor 10 for γ_E2_γ_W_ at the lowest reaction temperature. The desired reaction had a forward activity product of ~2.6, whereas the undesired reaction path showed a factor of ~1.8. In general, the product values, including water, showed the highest correction for activity in the liquid phase aldol condensation reaction. Also, the factor b, see above, reached values of 2.4 ± 0.3 kJ·mol^−1^ for the backward reaction involving *E*_2_. 

Taking a closer look at the core business of this manuscript, in silico experimental data were used in an in-house written parameter estimation routine, see Section 3.2, with the chemical reaction Equations (4) and (5), in which first step concentrations were used, i.e., all activity coefficients γ were set to unity by default. As expected, the variations in activation energy values for the two forward and backward reactions were not that pronounced, see entry ‘Without correction’ in Table 2, Table 3, Table 4 and Table 5, since a rather weak temperature dependency was observed for γ, the given test case of aldol condensation. On the other hand, huge deviations were observed in the pre-exponential factors. For example, with methanol as solvent, pre-exponential factor *k*_*2,∞*_ deviated ~31 times from the original value, see Table 2. The same parameter, *k*_*2,∞*_, showed significant deviation for solvents ethanol and n-propanol. n-Butanol gave a deviation of ~ 20. Parameter *k*_*4,∞*_ deviated ~10 times for ethanol as solvent, whereas deviations lower than factor 6 were obtained for n-propanol and n-butanol. 

In a second step, average activity coefficients were used, i.e., for the whole concentration profile only one value was implemented. The average values for γ were obtained using the in silico experimental data and point values for the activity coefficients, based on the experimental composition, acquired at equidistant time points; these average values distinctly differ from unity and range between 1 and 6. 

The results of the subsequent parameter estimation are reported in Table 2, Table 3, Table 4 and Table 5 with entry ‘With correction’. The reader can notice that most deviations were linked to the pre-exponential factors and that, after correction with the average activity coefficients, a better estimate for these factors was obtained. A slight improvement for the activation energies was witnessed. Especially, for *E_4_* with n-butanol as solvent a good improvement, going from 59.4 ± 6.2 kJ·mol^−1^ to 70.0 ± 2.0 kJ·mol^−1^ (true value was 70 kJ·mol^−1^) was observed. In the case of methanol the activation energy improved from 60.1 ± 4.4 kJ·mol^−1^ to 65.9 ± 3.5 kJ·mol^−1^. For ethanol and n-propanol no specific improvement was observed and the slight overestimation of *E_4_* can be explained by the earlier reported correction of 2.4 kJ·mol^−1^. 

**Figure 7:**
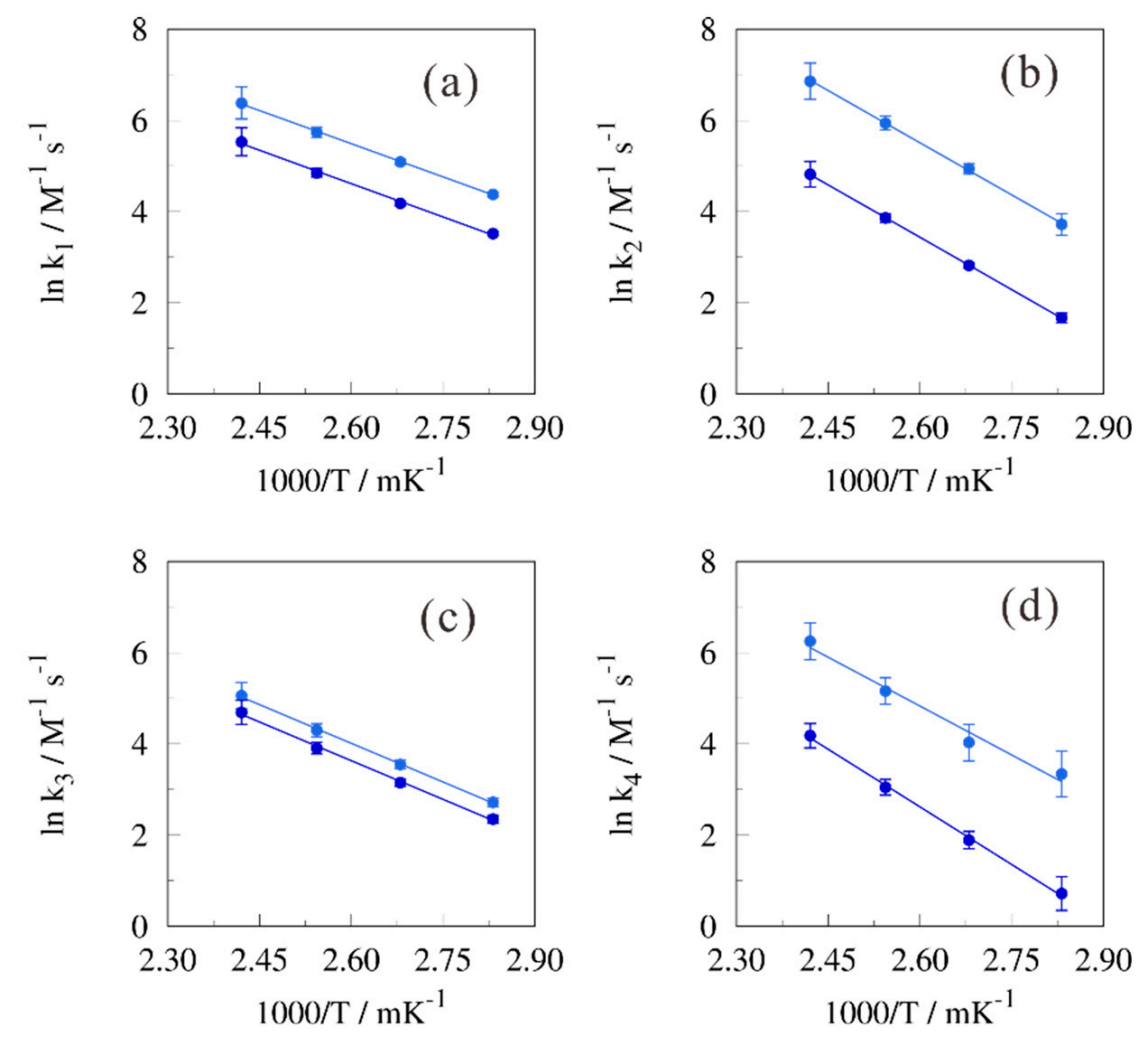
Arrhenius diagram for kinetic coefficients (**a**) *k1*; (**b**) *k2*; (**c**) *k3*; and (**d**) *k4* when n-butanol is used as solvent. Points and linear regression lines are for the (**─**,●) uncorrected data and (**─**,●) the corrected data using the average activity coefficients as reported in Table 5.

Arrhenius diagrams from the isothermal parameter estimation procedure with n-butanol as solvent are given in Figure 7. It was observed that, in the case when no correction was used for the activity coefficients, still very satisfactory Arrhenius diagrams were obtained. Including the correction, however, yielded a significantly different position with a smaller spread of the isothermal estimates. For the backward reaction coefficients, *k*_2_ and *k*_4_, the difference in position was the most pronounced. Similar diagrams and corresponding conclusions were obtained for the three other alcohol solvents.

The corresponding residual sum of squares (RSSQ) for the calculated concentrations is reported in Table 2, Table 3, Table 4 and Table 5. Although the estimated parameters were more in line with actual kinetics when the average value for γ was implemented, only a minimal difference in RSSQ has been noted. If these values are used in the conventional model discrimination tools, such as the F test or the so-called ‘likelihood ratio’ [3,25,26,27,28,29], the model with implementation and the model without would not be statistically different. However, it was shown that the ‘true’ kinetic parameters are better approached with the implementation of the average activity coefficients. This signifies a second caveat: In the interpretation of RSSQ for model discrimination, the inspection of the obtained kinetic parameters and the corresponding comparison to typical values is advised and the nonideal liquid properties should be incorporated. 

It has to be added that the choice of solvent in this in silico manuscript was only reflected in a variation of activity coefficients. If the choice of another solvent results in a real change in reaction mechanism [24], then of course the correction for the solvent type will not do the trick since fundamental chemical reactions or transition states are altered by this choice. 

So far, in silico data were presented and, therefore, it is of interest to link the reported results with some literature data. Some excellent references on experimental work can be mentioned [12,14,15,16]. In this report, two examples are addressed where alcohols were also used as solvent. 

First, when the selection of solvents as pure alcohol is enlarged to include binary mixtures of water-ethanol mixtures, Cueto et al. [27] described an enhancement of furfural-cyclopentanone aldol condensation as they observed that reaction coefficients increased for an increasing water content. A plausible reason for this is the stabilization of the formed enolate ion by water during the aldol condensation reaction. 

**Figure 8:**
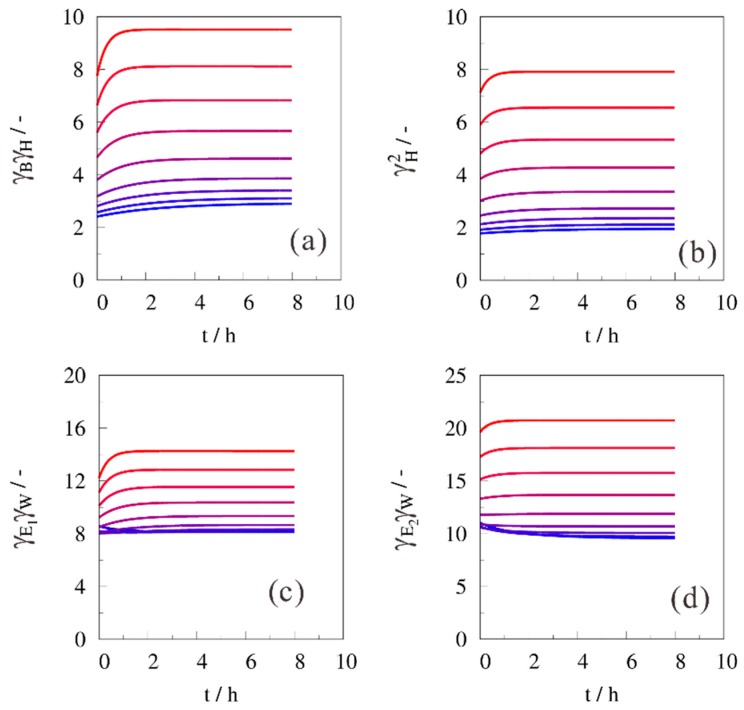
Activity coefficient products for (**a**) B*H; (**b**) H*H; (**c**) E_1_*W; and (**d**) E_2_*W with (*B*/*H*)_0_ = 2·mol·mol^−1^, T = 80 °C and solvent as ethanol. (EtOH/W)_0_ = (**─**) ∞ (pure EtOH), (**─**) 10, (**─**) 4, (**─**) 2, (**─**) 1, (**─**) 0.5, (**─**) 0.25, (**─**) 0.10, (**─**) 0 (pure water).

Starting from the same kinetic coefficient, *k_true_*, the forward reaction rate is written as *r* = *k_true_* × *a_B_* × *a_H_* or *r* = (*k_true_* × γ_B_ × γ_H_) × *C_B_* × *C_H_* and, hence, if authors report concentration dependencies for the description of their variations via continuity equations, the reported kinetic coefficient is the product of the true coefficient and the corresponding activity coefficients. Figure 8 shows the product of activity coefficients in both forward and backward reactions (4) and (5) for a varying (EtOH/W)_0_ ratio. Inspection of these results showed that as the ratio (EtOH/W)_0_ decreases, the product of activity coefficients significantly increases. This corresponds to the aforementioned experimental results and, hence, it is clear that the inclusion of activity coefficients in parameter estimation is a necessary requirement for correct data interpretation. It is also remarkable that these products can vary from a factor of ~2 to ~21. 

Secondly, solvent effects are also reported in liquid-phase hydrogenation reactions, e.g., Ramos et al. observed a pronounced solvent effect on the conversion of 4-(2-furyl)-3-buten-2-one over Pt/TiO_2_ catalyst regarding the use of protic and aprotic solvents [30]. The former solvents were varied as methanol, ethanol, 1- and 2-propanol, and pentanol, for which ethanol performed significantly better than methanol; and 1- and 2-propanol resulted in a marginal improvement of the conversion versus time profile. This is in line with the conclusion that ethanol had the best conversion and yield values towards the desired product in the present study. 

The major part of this work addresses alcohols as solvents. In order to investigate the general character of this work, a brief case study is mentioned where toluene and 1, 4-dioxane (non-polar), dimethylformamide (DMF) (polar aprotic), and ethanol and water (polar protic) were compared regarding activity coefficient values, and the corresponding conversion and product selectivity. 

**Figure 9:**
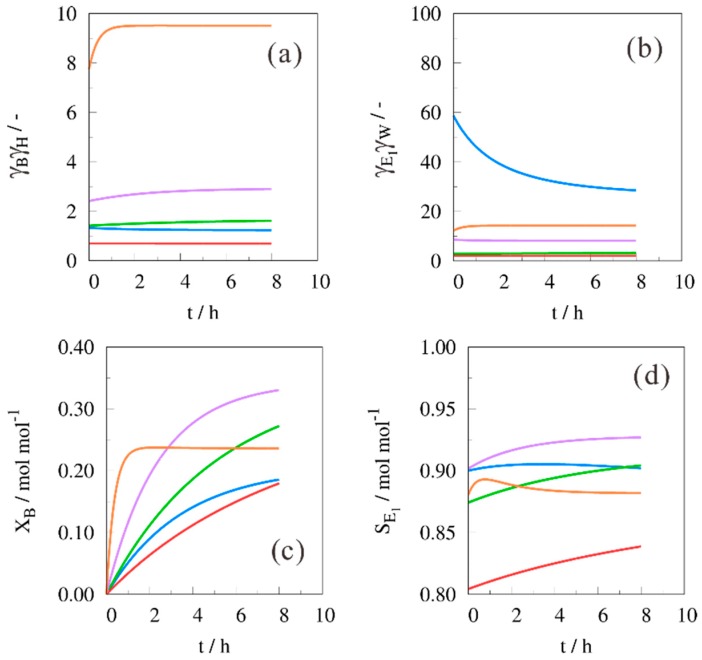
Activity coefficient products for (**a**) B*H; and (**b**) E_1_*W, present in reaction rate expression (4); (**c**) benzaldehyde conversion; and (**d**) selectivity towards product *E*_1_ with (*B*/*H*)_0_ = 2 mol·mol^−1^, T = 80 °C with the solvents (**─**) toluene, (**─**) 1,4-dioxane, (**─**) DMF, (**─**) ethanol and (**─**) water.

The results for the activity coefficient product B*H and E_1_*W are given in Figure 9a,b. For the forward reaction the order was: Water > ethanol > 1, 4-dioxane ≈ toluene > DMF. This explains the order of initial conversion profile, see Figure 9c. The equilibrium conversion when water was solvent was already established after 2 h; the other solvents required a longer reaction time. The conversion at 8 h reaction time can be ranked as ethanol > 1, 4-dioxane > water > toluene ≈ DMF. 

After ~2 h of reaction, ethanol performed the best to convert benzaldehyde, and together with the highest selectivity during the whole reaction time, see Figure 9d, these simulations confirm that ethanol is the best solvent to perform the given aldol condensation reaction. With DMF as solvent, the selectivity was significantly lower, compared to the others. 

As a closing remark, the author would like to state that the simulation results are not elaborated in terms of physical interpretation of the activity coefficients. This contribution merely indicates their effect on product distributions and corresponding parameter estimation results, starting from the same kinetics. In other words, sometimes different kinetic parameters are reported in literature for the same reaction with different solvents. This work indicates that it would be a good practice to use all experimental data simultaneously regarding parameter estimation, together with the varying nonideal liquid properties depending on the composition of the liquid phase. In this respect, the parameter estimation with a synchronous update of the activity coefficients yields to original parameter value, see Table 6, within 3% error. The runtime for this procedure was significantly higher than the case for average values. These results are omitted from the manuscript, since this was to be expected. 

## 3. Methods

### 3.1. Reaction Mechanism

According to Scheme 1, there are two reversible reactions included, see Equations (1) and (2), corresponding to the cross-condensation and the self-condensation reaction path:

(1) $$B+H⇄E_{1}+W$$

(2) $$2H⇄E_{2}+W$$

Benzaldehyde (*B*) and heptanal (*H*) react via cross aldol condensation into (2*E*)-2-benzylideneheptanal (*E*_1_), better known as ‘jasmin aldehyde’ and water (*W*). The self-condensation reaction gives the enal product (*Z*)-2-pentylnon-2-enal (*E*_2_). Some additional remarks on possible side reactions for the aldol condensation scheme are given in Appendix B. 

A so-called ‘activity coefficient’ is a factor used in thermodynamics to account for deviations from ideal behavior in a mixture of chemical substances. In an ideal mixture, the microscopic interactions between each pair of chemical species are the same so that the properties of the mixture can be expressed directly in terms of simple concentrations or partial pressures of the substances present. Deviations from ideality via molecular interactions in these mixtures are accommodated by modifying the concentration by an activity coefficient *γ*_i_ for compound *i*, *i* = 1…NC, see Equation (3), which can be conceptualized as a correction factor to ideal behavior and the activity is a better representation than the concentration is:

(3) $$a_{i}=\gamma_{i}C_{i}\;i\;=\;1\ldots \mathrm{NC}$$

The well-known UNIFAC group contribution model has been developed to estimate the nonelectrolyte activity in nonideal mixtures, i.e., it is based on activities rather than on concentrations [35,36,37,38,39]. The main feature is that in a group contribution model the real mixture is observed as a mixture of functional groups, rather than a mixture of compounds. 

The corresponding reaction rates for reactions (1) and (2) are given by Equations (4) and (5):

(4) $$r_{1}=k_{1}a_{B}a_{H}-k_{2}a_{E1}a_{W}$$

(5) $$r_{2}=k_{3}a_{H}^{2}-k_{4}a_{E2}a_{W}$$

Using expressions (4) and (5), the continuity Equations (CE), expressed in mol·L^−1^·s^−1^, for benzaldehyde (*B*), heptanal (*H*), jasmin aldehyde (*E*_1_), (*Z*)-2-pentylnon-2-enal (*E*_2_), and *W* (water) are given by Equations (6)–(10):

(6) $$\frac{dC_{B}}{dt}=-r_{1}$$

(7) $$\frac{dC_{H}}{dt}=-r_{1}-2r_{2}$$

(8) $$\frac{dC_{E1}}{dt}=r_{1}$$

(9) $$\frac{dC_{W}}{dt}=r_{1}+r_{2}$$

(10) $$\frac{dC_{E2}}{dt}=r_{2}$$

The corresponding initial conditions are given by Equations (11)–(13):

(11) $${\left(C_{B}\right)}_{t=0}=C_{B,0}$$

(12) $${\left(C_{H}\right)}_{t=0}=C_{H,0}$$

(13) $${\left(C_{E1}\right)}_{t=0}={\left(C_{E2}\right)}_{t=0}={\left(C_{W}\right)}_{t=0}=0$$

### 3.2. In-Silico Data and Parameter Estimation Procedure

The creation of in silico data is a common practice in literature when no experimental set-up is available [13,40]. Integration of CE (6)–(10), with updating the activity coefficients in every time step—see Section S.1 in the Supplementary Content—and implementing the initial conditions (11)–(13) gives the calculated responses for a reaction time of 8 h. Integration is performed via the Runge-Kutta method, see section S.2 in the Supplementary Content. 

Kinetic parameters are given in Table 6, containing ballpark values for the kinetic coefficients, based on existing literature for aldol condensation reactions [31,32,33,34]. Superposition of artificial Gaussian error is implemented at 10%, according to an earlier described procedure [40,41]. Error is implemented on calculated data every hour, giving 8 data points per response. 

Four sets of conditions are implemented, i.e., simulation sets 1 to 4 start from different solvents, methanol, ethanol, n-propanol, and n-butanol, with 3 initial concentration ratios: (*B*/*H*)_0_ = 2, 1, and 0.5 mol·mol^−1^. Under normal conditions an excess of heptanal is to be avoided, otherwise the self-condensation reaction byproduct results in low yields towards the industrially important jasmin aldehyde. For the determination of the kinetics, on the other hand, it is important to vary the concentrations as such so that the influence of this side reaction can be studied with significant detail. To calculate the initial concentrations, the initial number of moles are converted into a total volume. To this end, temperature-dependent density values are adopted [42]. 

In total, as a result of 5 responses (*B*, *H*, *E*_1_, *W*, and *E*_2_), 8 data points per response, and 3 sets of initial conditions, 120 data points are taken along the estimation procedure for every temperature level in each simulation set. Using the in silico experimental data, kinetic parameters are estimated using the ODRpack routine [43]. Details of the regression can be found elsewhere [25,26]. 

For graphical purposes, the conversion and product distribution is calculated according to Equations (14) and (15):

(14) $$X_{k}=\frac{C_{k,0}-C_{k}}{C_{k,0}}\;\;\;\;\;\;\;\;\;\;\;k\;=\;B,\;H$$

(15) $$S_{k}=\frac{C_{k}-C_{k,0}}{\left(C_{E1}-C_{E1,0}\right)+\left(C_{E2}-C_{E2,0}\right)}\;\;\;\;\;\;\;k\;=\;E_{1},\;E_{2}$$

The target of this study is to estimate the kinetic parameters of the given aldol condensation scheme, see reactions (4) and (5), with and without correction for the activity coefficients. In the case of the correction, average activity coefficients are used. The rationale behind this is that not every user has the simulation and parameter estimation code with synchronous update. Average activity coefficients are obtained using the in silico experimental data points, i.e., 9 activity coefficients are averaged. In reality, the application of this average, using the composition experimental data, corresponds to a minimal computational input of the user. 

Cueto et al. reported the application of binary water-ethanol mixtures as solvent for the improvement of furfural-cyclopentanone aldol condensation [27]. In order to investigate the influence of water as solvent on the given jasmin aldol condensation reaction, simulations with (EtOH/W)_0_ = 0 (pure water), 0.10, 0.25, 0.5, 1, 2, 4, 10, ∞ (pure ethanol) and (*B*/*H*)_0_ = 2 mol·mol^−1^ at T = 80, 110, and 140 °C were performed. 

## 4. Conclusions

In this work, the important industrial aldol condensation of benzaldehyde and heptanal was used to showcase the importance of activity coefficients in organic liquid phase reactions and the corresponding parameter estimation. 

It has been shown that omitting activity coefficients in parameter estimation procedures for liquid phase reactions has a distinct influence on the specific parameter values. A simple method, using average values for activity coefficients, was successfully proposed to generate acceptable parameter estimates. However, that a synchronous update gives the best results comes at a higher computational cost and increasing complexity of the applied computer programs. 

A second major conclusion is that the implementation of activity coefficients revealed distinct differences in conversion, product distribution, and selectivity. In the showcase, it appears that ethanol has the best solvent properties for the given aldol reaction. 

## Figures and Tables

**Table 1:** Activity coefficient products for the reactions (4) and (5) in the case of ethanol as solvent. ‘B*H’ indicates the product of the activity coefficients for the forward reaction of benzaldehyde and heptanal; other indications are similar. (*B*/*H*)_0_ in mol·mol^−1^.

(B/H)_0_	T (In °C)	B*H	H*H	E_1_*W	E_2_*W
2	80	2.752 ± 0.048	1.900 ± 0.022	8.251 ± 0.107	9.947 ± 0.049
	100	2.740 ± 0.045	1.903 ± 0.022	7.996 ± 0.104	9.537 ± 0.048
	120	2.680 ± 0.042	1.885 ± 0.021	7.726 ± 0.098	9.182 ± 0.048
	140	2.603 ± 0.039	1.856 ± 0.021	7.450 ± 0.093	8.836 ± 0.047
1	80	2.722 ± 0.069	1.763 ± 0.036	8.101 ± 0.152	9.155 ± 0.050
	100	2.720 ± 0.064	1.774 ± 0.035	7.850 ± 0.148	8.788 ± 0.048
	120	2.649 ± 0.058	1.756 ± 0.033	7.574 ± 0.140	8.479 ± 0.048
	140	2.556 ± 0.054	1.725 ± 0.032	7.295 ± 0.132	8.185 ± 0.048
0.5	80	2.676 ± 0.080	1.669 ± 0.045	8.097 ± 0.180	8.811 ± 0.051
	100	2.683 ± 0.074	1.686 ± 0.043	7.838 ± 0.177	8.451 ± 0.050
	120	2.610 ± 0.067	1.669 ± 0.040	7.553 ± 0.168	8.155 ± 0.049
	140	2.515 ± 0.062	1.646 ± 0.041	7.267 ± 0.155	7.910 ± 0.049

**Table 2:** Parameter estimates for set 1 (methanol as solvent). For the pre-exponential factors, the deviation is indicated between brackets.

Parameter	Unit	Without Correction	With Correction
*k* *1,∞*	M^−1^·s^−1^	(1.36 ± 0.01) × 10^4^ (1.70)	(7.62 ± 0.12) × 10^3^ (1.05)
*E*_1_	kJ·mol^−1^	40.8 ± 0.6	40.0 ± 0.9
*k*_*2,∞*_	s^−1^	(4.36 ± 0.16) × 10^7^ (31.4)	(4.71 ± 0.10) × 10^6^ (3.39)
*E*_2_	kJ·mol^−1^	66.4 ± 2.4	64.2 ± 1.4
*k*_*3,∞*_	M^−1^·s^−1^	(2.89 ± 0.02) × 10^4^ (1.25)	(2.84 ± 0.03) × 10^4^ (1.27)
*E*_3_	kJ·mol^−1^	46.8 ± 0.5	47.3 ± 0.6
*k*_*4,∞*_	s^−1^	(4.44 ± 0.26) × 10^6^ (3.13)	(4.91 ± 0.23) × 10^6^ (2.85)
*E*_4_	kJ·mol^−1^	60.1 ± 4.4	65.9 ± 3.5
RSSQ	μM^2^	0.470	0.342

**Table 3:** Parameter estimates for set 2 (ethanol as solvent). For the pre-exponential factors, the deviation is indicated between brackets.

Parameter	Unit	Without Correction	With Correction
*k* *1,∞*	M^−1^·s^−1^	(1.69 ± 0.01) × 10^4^ (2.11)	(1.01 ± 0.01) × 10^4^ (1.26)
*E*_1_	kJ·mol^−1^	39.6 ± 0.3	41.0 ± 0.5
*k*_*2,∞*_	s^−1^	(1.32 ± 0.02) × 10^7^ (9.54)	(3.82 ± 0.04) × 10^6^ (2.75)
*E*_2_	kJ·mol^−1^	61.2 ± 0.8	63.8 ± 0.7
*k*_*3,∞*_	M^−1^·s^−1^	(8.31 ± 0.11) × 10^4^ (2.30)	(5.35 ± 0.09) × 10^4^ (1.48)
*E*_3_	kJ·mol^−1^	49.1 ± 0.8	49.4 ± 1.1
*k*_*4,∞*_	s^−1^	(1.32 ± 0.04) × 10^8^ (9.52)	(4.06 ± 0.12) × 10^7^ (2.92)
*E*_4_	kJ·mol^−1^	70.8 ± 2.8	73.9 ± 2.5
RSSQ	μM^2^	0.298	0.287

**Table 4:** Parameter estimates for set 3 (n-propanol as solvent). For the pre-exponential factors, the deviation is indicated between brackets.

Parameter	Unit	Without Correction	With Correction
*k* *1,∞*	M^−1^·s^−1^	(1.87 ± 0.04) × 10^4^ (2.34)	(1.14 ± 0.03) × 10^4^ (1.42)
*E*_1_	kJ·mol^−1^	40.0 ± 1.1	41.4 ± 1.5
*k*_*2,∞*_	S^−1^	(1.31 ± 0.01) × 10^7^ (9.44)	(3.48 ± 0.04) × 10^6^ (2.51)
*E*_2_	kJ·mol^−1^	61.1 ± 0.6	63.5 ± 0.8
*k*_*3,∞*_	M^−1^·s^−1^	(8.72 ± 0.14) × 10^4^ (2.41)	(5.94 ± 0.07) × 10^4^ (1.65)
*E*_3_	kJ·mol-1	49.6 ± 1.0	49.8 ± 0.7
*k*_*4,∞*_	s^−1^	(7.27 ± 0.19) × 10^7^ (5.24)	(1.39 ± 0.05) × 10^7^ (1.00)
*E*_4_	kJ·mol^−1^	69.0 ± 2.1	70.5 ± 2.7
RSSQ	μM^2^	0.276	0.265

**Table 5:** Parameter estimates for set 4 (n-butanol as solvent). For the pre-exponential factors, the deviation is indicated between brackets.

Parameter	Unit	Without Correction	With Correction
*k* *1,∞*	M^−1^·s^−1^	(1.98 ± 0.03) × 10^4^ (2.48)	(8.98 ± 0.27) × 10^3^ (1.12)
*E*_1_	kJ·mol^−1^	40.3 ± 0.7	40.5 ± 1.6
*k*_*2,∞*_	s^−1^	(2.72 ± 0.03) × 10^7^ (19.6)	(3.79 ± 0.02) × 10^6^ (2.73)
*E*_2_	kJ·mol^−1^	63.3 ± 0.7	63.6 ± 0.3
*k*_*3,∞*_	M^−1^ s^−1^	(3.69 ± 0.07) × 10^4^ (1.02)	(2.56 ± 0.06) × 10^4^ (1.41)
*E*_3_	kJ·mol^−1^	47.0 ± 1.0	49.8 ± 0.7
*k*_*4,∞*_	s^−1^	(4.06 ± 0.03) × 10^6^ (3.43)	(1.24 ± 0.03) × 10^7^ (1.12)
*E*_4_	kJ·mol^−1^	59.4 ± 6.2	70.0 ± 2.0
RSSQ	μM^2^	0.201	0.197

**Table 6:** Ballpark values for the kinetic coefficients of the reactions given in Scheme 1.

Kinetic Coefficient	Unit	Reported Range	Ballpark Value
*k* *1,∞*	M^−1^·s^−1^	8.2 × 10^3^ [31]	8.0 × 10^3^
*E*_1_	kJ·mol^−1^	20–40 [32], 48.5 [31], 39.7–49.9 [33]	40
*k*_*2,∞*_	s^−1^		1.4 × 10^6^
*E*_2_	kJ·mol^−1^		60
*k*_*3,∞*_	M^−1^·s^−1^	1.35 × 10^3^–1.0 × 10^6^ [34]	3.6 × 10^4^
*E*_3_	kJ·mol^−1^	42.7–53.8 [34]	48
*k*_*4,∞*_	s^−1^		1.4 × 10^7^
*E*_4_	kJ·mol^−1^		70

## Supplementary Materials

The following are available online at https://www.mdpi.com/1422-0067/20/15/3819/s1, Supplementary materials regarding the UNIFAC method and Runge-Kutta scheme for numerical integration are uploaded with this work. Customized files or calculation of activity coefficients in liquid phase reactions can be obtained upon personal request at the author’s mail address.

## Abbreviations

B	benzaldehyde
CE	continuity equation
DMF	dimethylformamide
E_1_, E_2_	end product 1 and 2 (jasmin aldehyde and (*Z*)-2-pentylnon-2-enal)
GC-MS	gas chromatography mass spectrometry
H	heptanal
HPLC	high pressure liquid chromatography
NC	number of components
ODR	orthogonal distance regression
RSSQ	residual sum of squares
S	selectivity (mol·mol^−1^)
t	reaction time (dep.)
T	temperature (°C)
UNIFAC	UNIQUAC (universal quasichemical) functional-group activity coefficients
W	water
X	conversion (mol·mol^−1^)
*	‘*A***B*’ signifies the product of the activity coefficients for compound *A* and *B*

## Appendix A

Table A1 lists some reported conditions for the conversion of heptanal and heptanal under various conditions. If available, conversion and yield values are reported in this data collection.

It can be observed that industrial conditions often include catalytic systems, whereas this work—for the sake of interpretation and feasibility—considered homogeneous reactions with a basic catalyst such as NaOH.

**Table A1:** Reported experimental conditions for jasmin aldehyde production. B (benzaldehyde), (H) heptanal, S (selectivity), t (reaction time), T (temperature), X (benzaldehyde conversion).

Entry	Conditions	Catalyst	X (%)	S (%)	Ref.
1	B (15.8 mmol), H (7.9 mmol), *t* = 1 h, T = 125 °C	L-proline (40 mol%) + benzoic acid	99	96	[14]
2	p-chlorobenzaldehyde (0.5 mmol), isobutyraldehyde (dep.), Cs_2_O_3_ as base (10 mol%), solvent THF or xylene (5 mL)	N-heterocyclic carbene catalyst (10 mol%)	^(1)^	^(1)^	[15]
3	aliphatic and aromatic aldehydes ^(1)^	Ti(OR)_4_	^(1)^	^(1)^	[16]
4	*B* (7–79 mmol), *H* (2–31.6 mmol), *t*_max_ = 6 h, T = 100–180 °C	modified chitosan	^(2)^	^(2)^	[12]
5	n-heptanal (2.28 g) dropwise (during 30 min) in benzaldehyde (2.12 g), *t* = 3 h	K_2_CO_3_ (2.76 g) and benzyltriethylammonium chloride as phase transfer catalyst (2.5 g) in CH_2_Cl_2_	^(3)^	80	[17]
6	B (336 mmol), H (33.6 mmol), *t* = 2 h, T = 150 °C	Al-MCM-41 supported MgO (0.2 g)	9.4–96.7	40.7–56.2	[44]
7	B and H^(4)^, *t* = 15 min, T = 120 °C	pyrrolidine (30 mol%)	^(3)^	93	[45]
8	(*B/H*)_0_ = 2/1, T = 120 °C, solvent = DMF	MgO	94	68	[46]
9	benzaldehyde with C3–C8 linear aldehydes	K_2_CO_3_/Al_2_O_3_, KOH/Al_2_O_3_	^(1)^	^(1)^	[47]
10	(*B/H*)_0_ = 2/1, T = 80–140 °C, *t* < 7 h	Zn modified mixed Mg/Al Oxides	^(1)^	^(1)^	[48]
11	(*B/H*)_0_ = 2/1, T = 100 °C, no solvent	industrially prepared Mg–Al mixed oxides	<70	<66	[49]

^(1)^ Number of conditions too numerous to be listed in a concise way; ^(2)^ Only reaction rates (mmol h^−1^ g_cat_^−1^) were reported; ^(3)^ Not reported; ^(4)^ More examples of aldehydes in cross condensation reaction are reported in reference [45].

## Appendix B

Reaction (1) depicts the reaction of benzaldehyde (*B*) and heptanal (*H*) react via cross aldol condensation into (2*E*)-2-benzylideneheptanal (*E*_1_), better known as ‘jasmin aldehyde’ and water. The intermediate β-hydroxy carbonyl compound, 2-(hydroxy(phenyl)methyl)-heptanal, was considered to be present in very low concentration (water elimination occurs very fast), and therefore, it was omitted from the scheme. Reaction (2) gives the self-condensation reaction, where the intermediate β-hydroxy carbonyl compound 4-hydroxy-2-pentyl-nonanal was also omitted for the same reasoning.

Three additional remarks can be made on the given aldol reaction mechanism in Scheme 1 in Section 3.1. First, the so-called ‘Cannizzaro reaction’ is worthwhile mentioning: This reaction describes the disproportionation of a nonenolizable aldehyde, such as benzaldehyde, into a primary alcohol and a carboxylic acid. In this manuscript, benzyl alcohol and benzoic acid could be formed from benzaldehyde. For the sake of simplicity regarding the proposed research questions this option was left out. Literature data suggest that this reaction path only exists under highly basic conditions, but as Comisar and Savage point out a zero value for the corresponding kinetic coefficient in the reaction network containing the Cannizzaro reaction, the choice to leave it out was justifiable [32].

Secondly, the so-called ‘Tischenko reaction’ was reported earlier for aldol condensation reactions [50]. This reaction is a disproportionation reaction that allows the preparation of esters from two equivalents of an aldehyde. It was discarded in order not to overload the presented results.

Thirdly, the Murzon group reports that all reactions for the aldol condensation of cyclopentanone and valeraldehyde are irreversible [51]. Typical university textbooks report that the aldol condensation, giving the β-hydroxy aldehyde, is reversible and it is followed by irreversible water elimination [1,2]. On the other hand, general organic chemistry sources report that both are reversible [52] and, hence, for the sake of generality in the simplified scheme, both reactions were considered to be reversible.
