# Supplementary material for: Activity Coefficients for Liquid Organic Reactions: Towards a Better Understanding of True Kinetics with the Synthesis of Jasmin Aldehyde as Showcase
Source: Int J Mol Sci. 2019 Aug 5;20(15):3819. doi: 10.3390/ijms20153819 (PMC6695740; doi:10.3390/ijms20153819)
Supplement: Supplementary file 1 [file ijms-20-03819-s001.pdf]

S. Supplementary content

Section S.1 gives the governing equations for the UNIFAC method, taking into account nonidealities for liquid mixtures. Section S.2 contains the details on the 4th order Runge-Kutta integration scheme.

S.1. UNIFAC method

The UNIFAC group contribution model has been developed to estimate the non-electrolyte activity in non-ideal mixtures, i.e., it is based on activities rather than on concentrations [1-5]. In a group contribution model the real mixture is observed as a mixture of functional groups, rather than a mixture of compounds.

A so-called 'activity coefficient' is a factor used in thermodynamics to account for deviations from ideal behaviour in a mixture of chemical substances. In an ideal mixture, the microscopic interactions between each pair of chemical species are the same so that the properties of the mixture can be expressed directly in terms of simple concentrations or partial pressures of the substances present. Deviations from ideality via molecular interactions in these mixtures are accommodated by modifying the concentration by an activity coefficient γ_i for compound i, i = 1…NC, which can be conceptualized as a correction factor to ideal behavior and the activity is a better representation than the concentration is:

$$a_i = \gamma_i C_i \qquad \text{i = 1…NC} \qquad \text{(S-1)}$$

The UNIFAC model has two contributions for the activity coefficient of the compounds in the system, see Eq. (S-2):

$$\ln \gamma_i = \ln \gamma_i^C + \ln \gamma_i^r \qquad \text{i = 1…NC} \qquad \text{(S-2)}$$

In the UNIFAC model, there are three main parameters required in order to determine the activity

for each compound. Firstly there are the group surface area and volume contributions, obtained from the Van der Waals surface area and volumes – these parameters depend purely upon the individual functional groups on the host molecules. Thirs, there are the binary interaction parameters, which are related to the interaction energy U_i of molecular pairs. These parameters must be obtained either through experiments, via experimental data fitting or molecular simulations.

First, the so-called 'combinatorial part' of the activity coefficient is contributed by several terms in Eq. (S-3):

$$\ln\gamma_i^C = \ln\frac{\varphi_i}{x_i} + \frac{z}{2}q_i\ln\frac{\theta_i}{\varphi_i} + L_i - \frac{\varphi_i}{x_i}\cdot\sum_{j=1}^{n}x_jL_j \qquad i = 1\ldots NC \qquad \text{(S-3)}$$

θ_i and φ_i are the molar weighted segment and area fractional components for the compound i in the total system of n compounds; L_i is a compound parameter They are defined in Eqs. (S-4) to (S-6) with i = 1…NC:

$$\theta_i = \frac{x_iq_i}{\sum_{j=1}^{NC}x_jq_j} \qquad \text{(S-4)}$$

$$\varphi_i = \frac{x_ir_i}{\sum_{j=1}^{NC}x_jr_j} \qquad \text{(S-5)}$$

$$L_i = \frac{z}{2}\left(r_i - q_i\right) - \left(r_i - 1\right) \qquad \text{(S-6)}$$

q_i and r_i are calculated from the group surface area and volume contributions, see Table S1, taking into account the occurrence of the corresponding functional groups for each compound i, see Eqs. (S-7) and (S-8) with i = 1…NC:

$$r_i = \sum_{k=1}^{NG} \nu_k^{(i)} R_k \qquad \text{(S-7)}$$

$$q_i = \sum_{k=1}^{NG} \nu_k^{(i)} Q_k \qquad \text{(S-8)}$$

The value for z in Eqs. (S-3) and (S-6) is the coordination number of the system. The present UNIFAC model is relatively insensitive to this parameter and it is frequently set equal to the value 10.

Second, the so-called 'residual part' is to be considered. This contribution is due to interactions between groups present in the system. The residual component of the activity coefficient for compound i, containing n unique functional groups, is calculated via Eq. (S-9) – k is the index accounting for the individual functional groups:

$$\ln \gamma_i^r = \sum_{k=1}^{n} \nu_k^{(i)} \left(\ln \Gamma_k - \ln \Gamma_k^{(i)} \right) \qquad i = 1 \ldots NC \qquad \text{(S-9)}$$

$\Gamma_k^{(i)}$ represents the activity of an isolated group in a solution consisting only of molecules of type i. The formulation of the residual activity ensures that the condition for the limiting case of a single molecule in a pure component solution, the activity is equal to the corresponding concentration. Γ_k is calculated via Eq. (S-10), where θ_ℓ is the summation of the area fraction of group ℓ over all the different groups, see Eq. (S-11). $\Psi_{\ell,k}$ is the group interaction parameter and it is a measure of the interaction energy between groups, see Eq. (S-12):

$$\ln \Gamma_k = Q_k \left(1 - \ln \sum_{\ell=1}^{NG} \theta_\ell \Psi_{\ell,k} - \sum_{\ell=1}^{NG} \frac{\Psi_{k,\ell}\theta_\ell}{\sum_{m=1}^{NG} \theta_m \Psi_{m,\ell}} \right) \qquad k = 1 \ldots NG \qquad \text{(S-10)}$$

$$\theta_\ell = \frac{Q_\ell X_\ell}{\sum_{m=1}^{NG} Q_m X_m} \qquad \ell = 1 \ldots NG \qquad \text{(S-11)}$$

$$\Psi_{\ell,k} = \exp\left(-\frac{U_{\ell,k} - U_{k,\ell}}{RT}\right) \qquad \text{(S-12)}$$

X_n is the group mole fraction, which is the number of groups n in the solution divided by the total number of groups, see Eq. (S-13):

$$X_\ell = \frac{\sum_{i=1}^{NC} \nu_\ell^{(i)} x_i}{\sum_{j=1}^{NG}\sum_{i=1}^{NC} \nu_j^{(i)} x_i} \qquad \ell = 1 \ldots NG \qquad \text{(S-13)}$$

$U_{a,b}$ represents the interaction energy between groups a and b, in J mol^{-1}, and R and T are the universal gas constant and the temperature in K. It has to be noted that $U_{a,b} \neq U_{b,a}$ so that a on-reflexive parameter is $a_{a,b}$ is used – normally this parameter is tabulated, see Eq. (S-14):

$$\Psi_{\ell,k} = \exp\left(-\frac{a_{\ell,k}}{RT}\right) \qquad \text{(S-14)}$$

$a_{a,b}$ represents the net interaction energy between then given groups a and b and it has the unusual unit K. The applied values are mentioned in Table S2.

S.2. Runge-Kutta method

In order to avoid that the reader should dive into books filled with difficult and depressing mathematics, the author would like to summarize here the required equations for the implementation of the so-called ‘4th order Runge-Kutta method’ for a set of coupled differential equations (S-15) with initial conditions (S-16) with i = 1…5 and $(\underline{y})_i = y_i(x)$:

$$\frac{dy_i}{dx} = f_i\left(x, \underline{y}(x)\right) \tag{S-15}$$

$$y_i\left(x_0\right) = y_{i,0} \tag{S-16}$$

The 4th order RK scheme is an integration scheme with higher precision than the frequently used Euler method (1st order scheme). The iteration to go from step n to n+1 is governed by Eqs. (S-21) and (S-22) with intermediate calculations (S-17) to (S-20) with i = 1…5 and h is the integration step:

$$\left(\underline{k}\right)_i = k_i = h \cdot f_i\left(x_n, \underline{y}\left(x_n\right)\right) \tag{S-17}$$

$$\left(\underline{s}\right)_i = s_i = h \cdot f_i\left(x_n + \frac{h}{2}, \underline{y}\left(x_n\right) + \frac{1}{2}\underline{k}\right) \tag{S-18}$$

$$\left(\underline{l}\right)_i = l_i = h \cdot f_i\left(x_n + \frac{h}{2}, \underline{y}\left(x_n\right) + \frac{1}{2}\underline{s}\right) \tag{S-19}$$

$$\left(\underline{p}\right)_i = p_i = h \cdot f_i\left(x_n + h, \underline{y}\left(x_n\right) + \underline{l}\right) \tag{S-20}$$

$$x_{n+1} = x_n + h \tag{S-21}$$

$$\left(\underline{y}\right)_i = y_i\left(x_{n+1}\right) = y_i\left(x_n\right) + \frac{k_i + 2s_i + 2l_i + p_i}{6} \tag{S-22}$$

Table S1. Group contribution parameters for UNIFAC method (part 1) [1-4]. B = benzaldehyde, H = heptanal, E1 = jasmin aldehyde and E2 = (Z)-2-pentylnon-2-enal, W = water. (S1, S2, S3, S4) = (methanol, ethanol, n-propanol, n-butanol).

Nr.	Main group	Subgroup	R_k	Q_k	B	H	E1	E2	W	S1	S2	S3	S4
1	1	1a	0.9011	0.848		1	1	2			1	1	1
2	1	1b	0.6744	0.540		5	4	9			1	2	3
3	2	2d	0.8886	0.676			1	1					
4	3	3a	0.5313	0.400	5		5						
5	3	3b	0.3652	0.120	1		1						
6	5	5	1.0000	1.200							1	1	1
7	6	6	1.4311	1.432						1			
8	7	7	0.9200	1.400					1				
9	10	10	0.9980	0.948	1	1	1	1					

Table S2. Group: Interaction parameters $a_{i,j}$, (i,j) = (row, column), for the groups in the UNIFAC method (unit is K) [1-4]. Shaded areas indicate no interaction (same main group in UNIFAC method).

	1	1	2	3	3	5	6	7	10
1			86.02	61.13	61.13	986.5	697.2	1318	677.0
1			86.02	61.13	61.13	986.5	697.2	1318	677.0
2	-35.36	-35.36		38.81	38.81	524.1	787.6	270.6	448.8
3	-11.12	-11.12	3.446			636.1	637.5	903.8	347.3
3	-11.12	-11.12	3.446			636.1	637.5	903.8	347.3
5	156.4	156.4	457.0	89.6	89.6		-137.1	353.5	-203.6
6	16.51	16.51	-12.52	-50.0	-50.0	249.1		-181.0	306.4
7	300.0	300.0	496.1	362.3	362.3	-229.1	289.6		-116.0
10	505.7	505.7	56.3	23.39	23.39	529.0	-340.2	480.8	

References

[1] A. Fredeslund, R.L. Jones, J.M. Prausnitz, Group-contribution estimation of activity coefficients in nonideal liquid mixtures, AIChE J. 21 (1975) 1086-1099.

[2] H.K. Hansen, P. Rasmussen, A. Fredeslund, S. M., J. Gmehling, Vapor-liquid equilibria by UNIFAC group contribution. 5. Revision and extension, Ind. Eng. Chem. Res. 30 (1991) 2352-2355.

[3] R. Wittig, J. Lohmann, J. Gmehling, Vapor−liquid equilibria by UNIFAC group contribution. 6. Revision and extension, Ind. Eng. Chem. Res. 42 (2003) 183-188.

[4] C. Peng, M.N. Chan, C.K. Chan, The hygroscopic properties of dicarboxylic and multifunctional acids: masurements and UNIFAC predictions, Environ. Sci. Technol. 35 (2001) 4495-4501.

[5] M. Kaufman, Principles of Thermodynamics, Marcel Dekker, Inc., New York, 2002.
